# The combination of the error correction methods of GAFCHROMIC EBT3 film

**DOI:** 10.1371/journal.pone.0181958

**Published:** 2017-07-27

**Authors:** Yinghui Li, Lixin Chen, Jinhan Zhu, Xiaowei Liu

**Affiliations:** 1 School of Physics, Sun Yat-sen University, Guangzhou, Guangdong, People’s Republic of China; 2 State Key Laboratory of Oncology in South China, Sun Yat-sen University Cancer Center, Guangzhou, Guangdong, People’s Republic of China; North Shore Long Island Jewish Health System, UNITED STATES

## Abstract

**Purpose:**

The aim of this study was to combine a set of methods for use of radiochromic film dosimetry, including calibration, correction for lateral effects and a proposed triple-channel analysis. These methods can be applied to GAFCHROMIC EBT3 film dosimetry for radiation field analysis and verification of IMRT plans.

**Methods:**

A single-film exposure was used to achieve dose calibration, and the accuracy was verified based on comparisons with the square-field calibration method. Before performing the dose analysis, the lateral effects on pixel values were corrected. The position dependence of the lateral effect was fitted by a parabolic function, and the curvature factors of different dose levels were obtained using a quadratic formula. After lateral effect correction, a triple-channel analysis was used to reduce disturbances and convert scanned images from films into dose maps. The dose profiles of open fields were measured using EBT3 films and compared with the data obtained using an ionization chamber. Eighteen IMRT plans with different field sizes were measured and verified with EBT3 films, applying our methods, and compared to TPS dose maps, to check correct implementation of film dosimetry proposed here.

**Results:**

The uncertainty of lateral effects can be reduced to ±1 cGy. Compared with the results of Micke A et al., the residual disturbances of the proposed triple-channel method at 48, 176 and 415 cGy are 5.3%, 20.9% and 31.4% smaller, respectively. Compared with the ionization chamber results, the difference in the off-axis ratio and percentage depth dose are within 1% and 2%, respectively. For the application of IMRT verification, there were no difference between two triple-channel methods. Compared with only corrected by triple-channel method, the IMRT results of the combined method (include lateral effect correction and our present triple-channel method) show a 2% improvement for large IMRT fields with the criteria 3%/3 mm.

## Introduction

To ensure consistency of the medical prescription within the clinical tolerance, and safe fulfilment of that prescription during the treatment. It is necessary to perform quality assurance (QA) analyses for the devices and methods associated to radiotherapy treatment process, such as linear accelerator and treatment planning system [1]. Compared with an ionization chamber or semiconductor matrix, GAFCHROMIC EBT film dosimetry offers the advantages of high spatial resolution, near-tissue equivalence and weak energy dependence [2–5]. The dosimetric characterization of GAFCHROMIC EBT film has been demonstrated by Martina et al. [6], and it has been shown to be suitable for clinical dosimetry.

Published works [7–12] indicate that there are several artefacts that can limit the dose accuracy of GAFCHROMIC EBT film. These include effect related to darkening, temperature, scanner performance, film orientation, and film inhomogeneities. According to the investigation of Mack et al. [7], the darkening effect can be negligible when scanning at least 24 h after irradiation. The effect of temperature is reversible between 15°C and 25°C, and the effect is -0.02%/K. The effect of scanner performance is a lateral effect [8–10], and film inhomogeneity can cause disturbances [11]. Several studies showed that reducing these effects is critical for ensuring that GAFCHROMIC EBT film accurately reflects dose characteristics [13–19].

For scanner performance, several studies [20–23] demonstrated that charge-coupled device (CCD) scanners have a lateral effect (non-uniform lateral response of the scanner) in the orthogonal direction (perpendicular to the movement axis of the scanner lamp), and the lateral effect is dose and position dependent. Menegotti L et al. [21] corrected lateral effect by extracting parabolic correction factors at different dose levels. Poppinga D et al. [22] corrected it by fitting the OD curvatures of parabolas at different dose levels. Wen N et al. [23] built a scanner non-uniform response correction map by registering and comparing film doses to the reference ion chamber array-based dose map delivered with the same doses.

Film inhomogeneity also effects dose accuracy [11, 24, 25]. Micke A et al. [11] proposed a triple-channel method, which is based on extracting the relative thickness of the active layer and separating the dose-dependent part of the image from the disturbance map, and using this method, the accuracy of intensity-modulated radiation therapy (IMRT) plan verification was significantly improved. Mayer RR et al. [26] and Perez Azorin JF et al. [27] introduced two disturbances, one common to the three channels and one depending on the colour channel, to improve film uniformity.

GAFCHROMIC EBT films have been demonstrated to be capable of effectively validating 2D IMRT dose distributions in phantoms [11, 12, 25, 27, 28]. In addition, the application of the EBT3 films to the 3D dose distribution has also been demonstrated [4].

This study combines a set of methods for radiochromic film dosimetry, which is able to consider lateral non-uniform scanner response, calibration and film inhomogeneity for an effective use of this high-resolution dose detection system. The results for dosimetric characteristics measured using GAFCHROMIC EBT film can be applied to radiation field analyses and the verification of IMRT plans.

## Materials and methods

### Irradiation and scanning process

GAFCHROMIC EBT3 films with sheet dimensions of 20.32 × 25.40 cm^2^ were used in this study. To avoid discrepancies, films from the same batch (Lot No. 06051404) were used. The films were irradiated with 6-MV photons using a Varian Unique linear accelerator.

To analyse lateral effects, four EBT3 films were irradiated to four uniform dose levels, ranging from 0 to 372.5 cGy. Each film was placed in a multilayer 40 × 40 × 20 cm^3^ Solid Water phantom at a depth of 10 cm and a source-to-surface distance (SSD) of 90 cm. Each film was irradiated with a field 40 × 40 cm^2^ in size, and the dose values were measured using Matrixx array at the same depth for comparisons.

**Fig 1:**
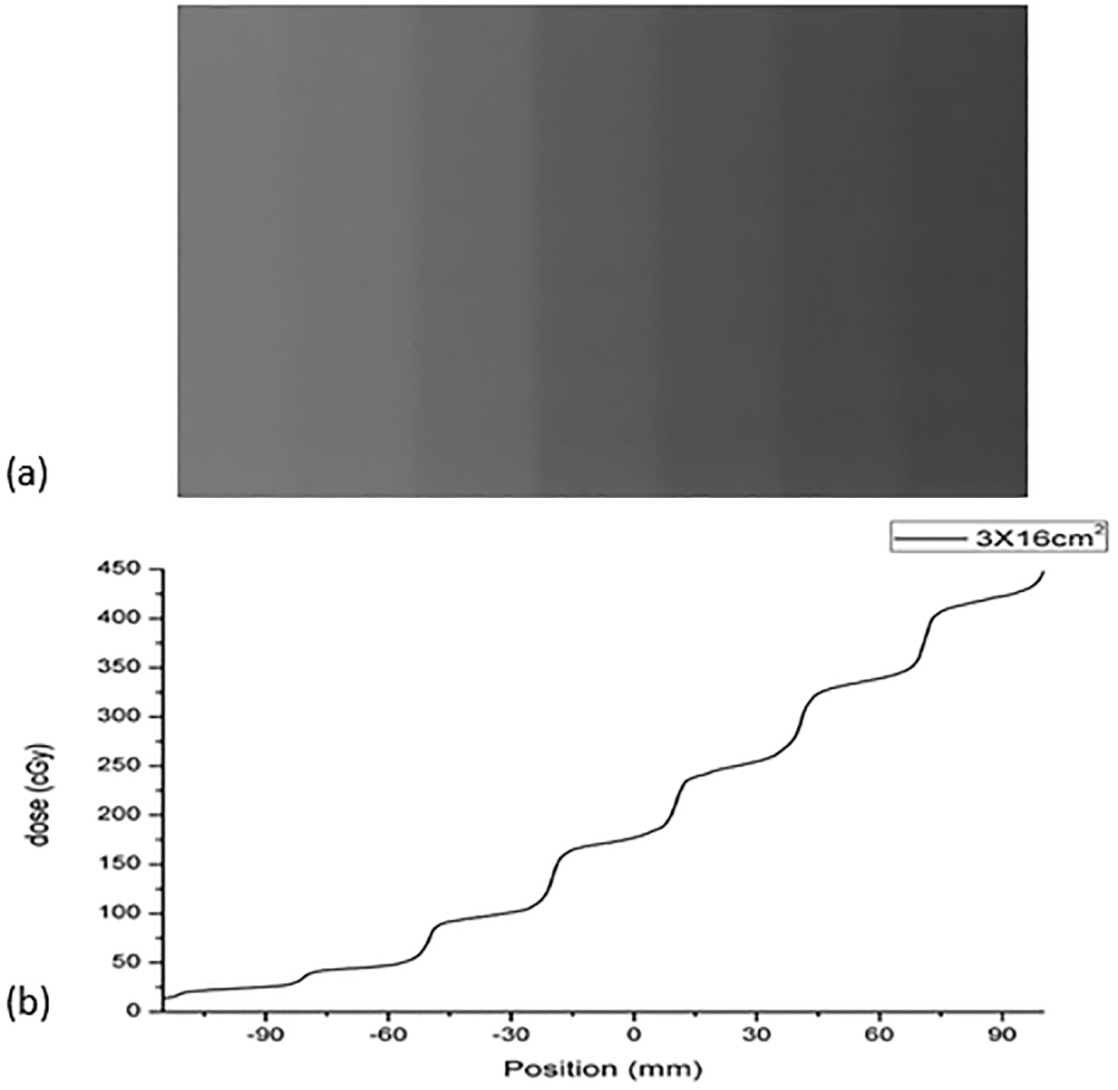
Scheme of the calibration method with seven strips (3.0 × 16 cm2).

To obtain the film calibration curve, the single film calibration method was used for fast dose calibration, as shown in Fig 1. The film was placed in a multilayer 40 × 40 × 20 cm^3^ Solid Water phantom at a depth of 10 cm and a SSD of 90 cm. To evaluate the proper width of the strips, two groups of strips with different widths (2.5 and 3.0 cm) were generated by irradiating the films with 6-MV photon beams. The calibration plans were designed in the Eclipse v.10.0 treatment planning system, and a total of ten and nine side-by-side static fields were generated for the 2.5- and 3.0-cm strips, respectively. Marginal strips were used not as calibration strips, but rather to ensure the uniformity of the strips on both sides. To reduce measurement uncertainty, each strip irradiation calibration method was performed three times, and the average OD values were used in the calibration. Moreover, to ensure the accuracy of the single-film calibration method, the square-field calibration method [29] was used as the standard to determine the proper strip size. Two EBT3 films were cut into eight equal pieces, and their orientation was marked on each piece. The pieces were exposed to 10 × 10-cm^2^ fields. After exposure, all pieces were joined with transparent tape as indicated by the marks. The calibration dose value was measured using a PTW UNIDOS electrometer with a 0.125-cm^3^ Semiflex ionization chamber (31010) at the centre point of each strip and square field. The ionization chamber and film were measured separately to maintain them at the same depth. For the measurement of strip dose values, all side-by-side fields were executed during the measurement of each centre point. In this way, the contribution of each field on the single film can be measured by the ionization chamber, including the contribution to scattering.

At least 24 h after exposure, all films were scanned within 30 min. The films were scanned and digitized using an Epson 10000XL flatbed scanner in transmission mode without colour correction and with a resolution of 72 dpi in 48-bit RGB (red, green and blue) mode (16 bits per channel, with pixel values in the range of 0–2^16^−1, the real value and its corresponding uncertainty is 65207±280), and the centres of the film and scanner were placed in a co-axial orientation. The data were saved as TIFF (tagged image file format) files. The films were positioned using a thin black plastic frame to ensure that they were all scanned in the same position, and the frame was not removed until all films were scanned. To reduce the effect of scanning temperature, the scanner was placed in an environment with a temperature of 20 ± 2°C.

All films were placed in the scanner with their long edge parallel to the scanning direction. Each film was scanned three times, and the result of the third scan was used for data analysis [30]. The dimensions of the scanned area were set to 19.4 × 24.6 cm^2^.

### Correction of lateral effects

**Fig 2:**
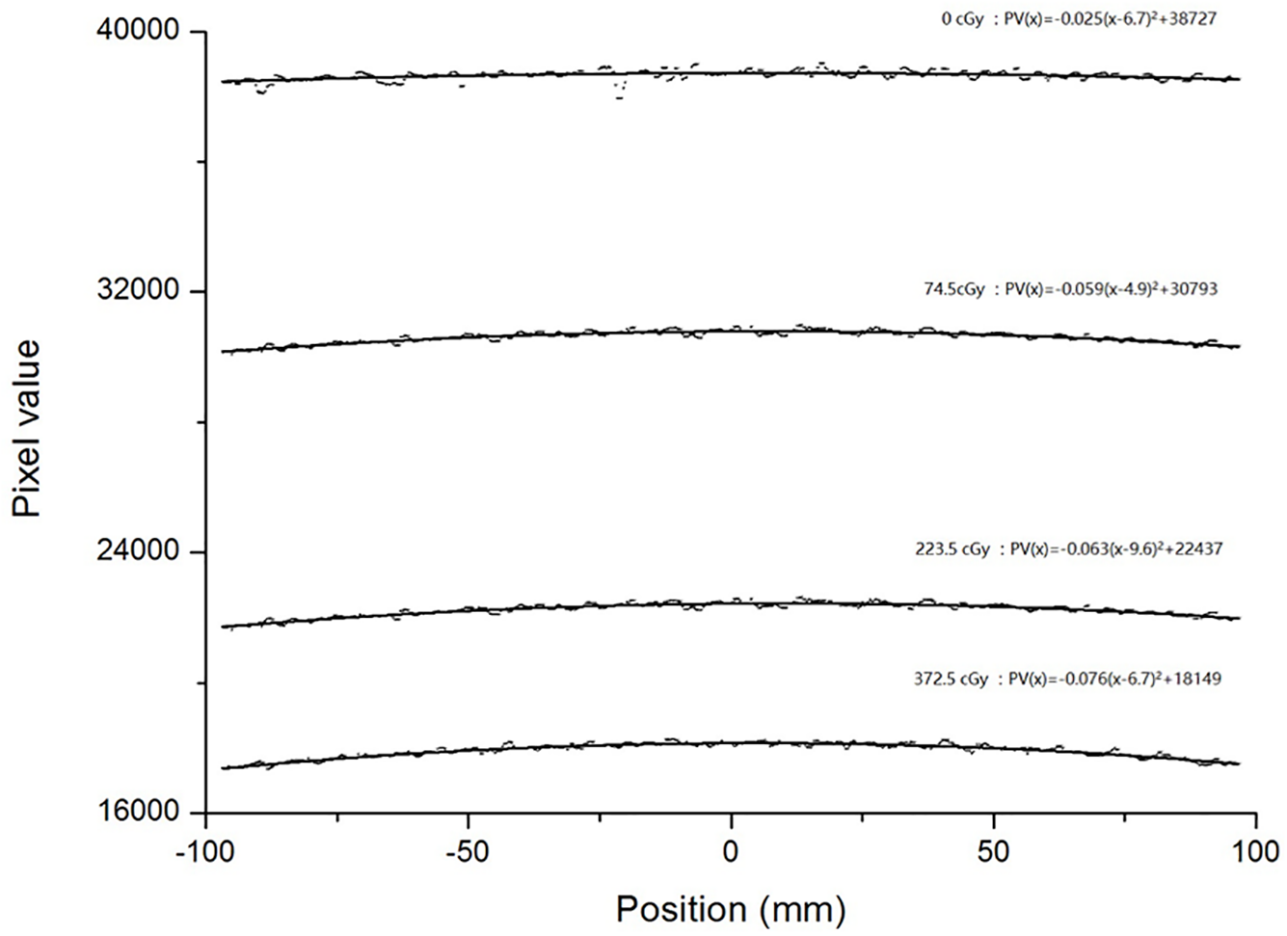
The scanned pixel values of the red channel and their second-order polynomial fits at different constant dose values.

The lateral effect of a scanner is position and dose dependent. As shown in Fig 2, for a constant dose, the tendency of the lateral effect is similar to a parabolic curve in the orthogonal direction (X axis) and can be fitted as a parabolic function:

$${PV}_{k}(x)={a_{k}(x-b_{k})}^{2}+c_{k},$$ (1)

where *k* represents the RGB channel, *x* represent the position coordinates, the pixel values *PV*_*k*_(*x*) were obtained by scanning the film at position x, and *a*_*k*_, *b*_*k*_ and *c*_*k*_ are fitting coefficients. The coefficient *a*_*k*_ is the curvature of the parabola. The parabolic vertex coordinates are (*b*_*k*_,*c*_*k*_). According to the methods of Poppinga D et al. [22], the parabolic symmetry axis, *x* = *b*_*k*_, was found to be a same constant at different dose levels, and the relationship between *a*_*k*_ and *c*_*k*_ can be fitted as a quadratic function:

$$a_{k}=p_{k}+q_{k}c_{k}^{2}$$ (2)

where *p*_*k*_ and *q*_*k*_ are fitting coefficients. Therefore, *Eq* (1) can be written as

$${PV}_{k}(x)={\left(p_{k}+q_{k}c_{k}^{2})(x-b_{k}\right)}^{2}+c_{k}$$ (3)

where *PV*_*k*_(*x*) is determined by scanning the film at position x, and *c*_*k*_, as the corrected pixel value (*PV*_*k*,*corr*_), can be obtained by solving Eq (3).

### Dose calibration function

The scanned optical density (OD) was defined as follows:

$${OD}_{k}={log}_{10}\left(\frac{65207}{{PV}_{k,corr}}\right).$$ (4)

The following exponential formula was used for dose calibration:

$$\bar{{OD}_{k}}=c_{k}-a_{k}∙e^{{-b}_{k}∙D},$$ (5)

where $\bar{{OD}_{k}}$ is the average OD of the central region (1 × 2 cm^2^) of each exposed strip, *D* is the absorbed dose measured by the ionization chamber and coefficients *a*_*k*_, *b*_*k*_ and *c*_*k*_ were obtained using a nonlinear least-squares fitting technique.

### Triple-channel dose analysis

A triple-channel film dosimetry method was proposed by Micke A et al. [11] based on the use of multiple colour channels to convert scanned images of radiochromic films into dose maps. According to this method, the scanned OD at any point is directly proportional to a dimensionless measure of the thickness of the active layer coated on the film:

$${OD}_{k}=u_{k}∙t,$$ (6)

where *u*_*k*_ is the absorption coefficient of the active layer, which varies with the absorbed dose, and *t* is the thickness of the active film layer. According to the manufacturer, EBT3 films consist of an active layer and two matte polyester substrates, and the absorption coefficient of the substrate does not vary with the absorbed dose. Thus, the scanned OD at any point can be divided into two components:

$${OD}_{k}={OD}_{net}+{OD}_{bg},$$ (7)

where *OD*_*net*_ is the net OD due to radiation exposure and *OD*_*bg*_ is the OD of the background film.

According to *Eqs* (6) and (7), the scanned OD in our method can be expressed as

$${OD}_{k}=u_{net,k}∙t+u_{bg,k}∙t_{f},$$ (8)

where *u*_*net*,*k*_ and *u*_*bg*,*k*_ are the absorption coefficients of the net OD and background, respectively; *t* is the thickness of the active layer, and *t*_*f*_ is the film thickness. When *t* = *t*_*f*_, *Eq* (8) becomes *Eq* (6). In *Eq* (5), $\bar{{OD}_{k}}$ is the average OD; thus, the corresponding thicknesses become the average thicknesses ($\bar{t}$ and $\bar{t_{f}}$):

$$\bar{{OD}_{k}}=u_{net,k}∙\bar{t}+u_{bg,k}∙\bar{t_{f}}.$$ (9)

When *D* = 0, the average OD is the background *OD*:

$$\bar{{OD}_{k}}=u_{bg,k}∙\bar{t_{f}}=c_{k}-a_{k}.$$ (10)

Thus, the average net *OD* can be written as follows:

$$\bar{{OD}_{net,k}}=u_{net,k}∙\bar{t}=a_{k}(1-e^{-b_{k}∙D}).$$ (11)

The scanned OD at any point can be written as

$${OD}_{k}=u_{net,k}∙\bar{t}∙\Delta t+u_{bg,k}∙\bar{t_{f}}∙\Delta t_{f}=a_{k}(1-e^{-b_{k}∙D})∙\Delta t+(c_{k}-a_{k})∙\Delta t_{f},$$ (12)

where $\Delta t=t/\bar{t}$ and ${\Delta t}_{f}=t_{f}/\bar{t_{f}}$ are the relative thicknesses of the active layer and film, respectively, and the absorbed dose at any point for each channel can be evaluated as follows:

$$D_{k}=-\frac{1}{b_{k}}∙ln\left(\frac{a_{k}∙\Delta t+(c_{k}-a_{k})∙\Delta t_{f}-{OD}_{k}}{a_{k}∙\Delta t}\right).$$ (13)

Because the thickness of the film transmitting all three types of light is the same at a pixel point, the relative thickness can be used as a bridge to relate the three single channels, differences among which can be minimized using the least-squares criterion:

$$\varphi (\Delta t,{\Delta t}_{f})={\left(D_{R}-D_{G}\right)}^{2}+{\left(D_{G}-D_{B}\right)}^{2}+{\left(D_{B}-D_{R}\right)}^{2}.$$ (14)

Using *Eq* (14), we can find a set of optimal Δ*t* and Δ*t*_*f*_ values to minimize the function *φ*(Δ*t*,Δ*t*_*f*_) and then substitute Δ*t* and Δ*t*_*f*_ into *Eq* (13) to calculate the dose value for each channel (*D*_*R*_, *D*_*G*_ and *D*_*B*_).

After being corrected by a triple-channel analysis, the average triple-channel dose is used as the measurement result:

$$D_{average}=\frac{D_{R}+D_{G}+D_{B}}{3}.$$ (15)

### Verification

To verify the improvement of our proposed triple-channel method, a 3 × 16 cm^2^ calibration film was corrected using both the proposed method and the method of Micke A et al. [11] individually, and the results were compared with those from a Matrixx 2D detector (IBA). To better illustrate the improvement of the triple-channel method, no nonlinear corrections were applied for this comparison.

To verify the validity of our combined method (include lateral effect correction and our present triple-channel method), a PDD and four off-axis ratios (OARs) were generated using a 6-MV Varian Unique linear accelerator following our method. The OAR is usually defined as the ratio of the dose at an off-axis point to the dose along the central beam axis at the same depth in a phantom. The results were compared with the data measured using a 0.13-cm^3^ ionization chamber (IBA CC13) in a 3D water tank. The field size of the PDD was 10 × 10 cm^2^, and the SSD was 100 cm. The film was irradiated at 300 MU (200 cGy at a 10-cm depth) and placed parallel to the beam axis in a multilayer 40 × 40 × 40-cm^3^ Solid Water phantom. The OAR results were measured at a depth of 10 cm and a SSD of 100 cm. The field sizes were 3 × 3, 5 × 5, 10 × 10 and 15 × 15 cm^2^, and MU was set to 200.

To evaluate the potential benefits of our combined method, small, medium and large IMRT fields were tested. A total of 18 IMRT plans (designed in Eclipse v.10.0 treatment planning system) with 6 small fields (< 10 × 10 cm^2^) for brain cancer, 6 medium fields (10 × 10 cm^2^–15 × 15 cm^2^) for lung cancer and 6 large fields (> 15 × 15 cm^2^) for Nasopharyngeal carcinoma (NPC) were irradiated. All fields were irradiated at 0 degree of gantry angle. The film dose results were analysed with our combined method and the method of Micke A et al. [11]. Film dose distributions were compared to TPS dose maps, to check correct implementation of film dosimetry proposed here. The gamma evaluation method was used for the evaluation of agreement between the film and TPS dose distributions. The criteria of γ 2D(3% dose difference/3 mm distance), γ 2D(2%/2 mm) and γ 2D(1%/1 mm) were evaluated in this study. Point doses less than 10% of the maximum dose were not considered.

## Results

### Correction of lateral effects

**Fig 3:**
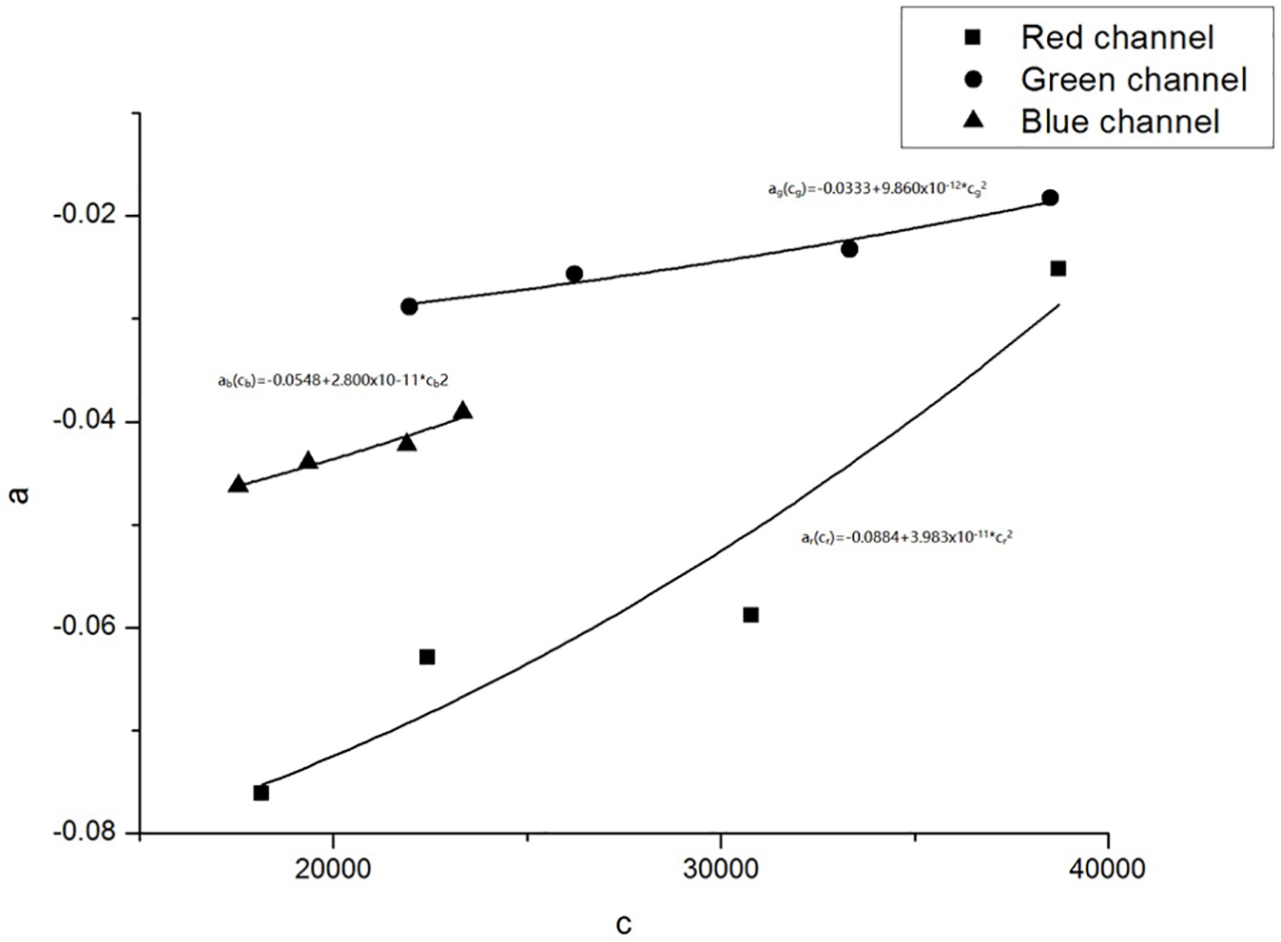
The relationships between the parameters a and c in the RGB channels.

**Fig 4:**
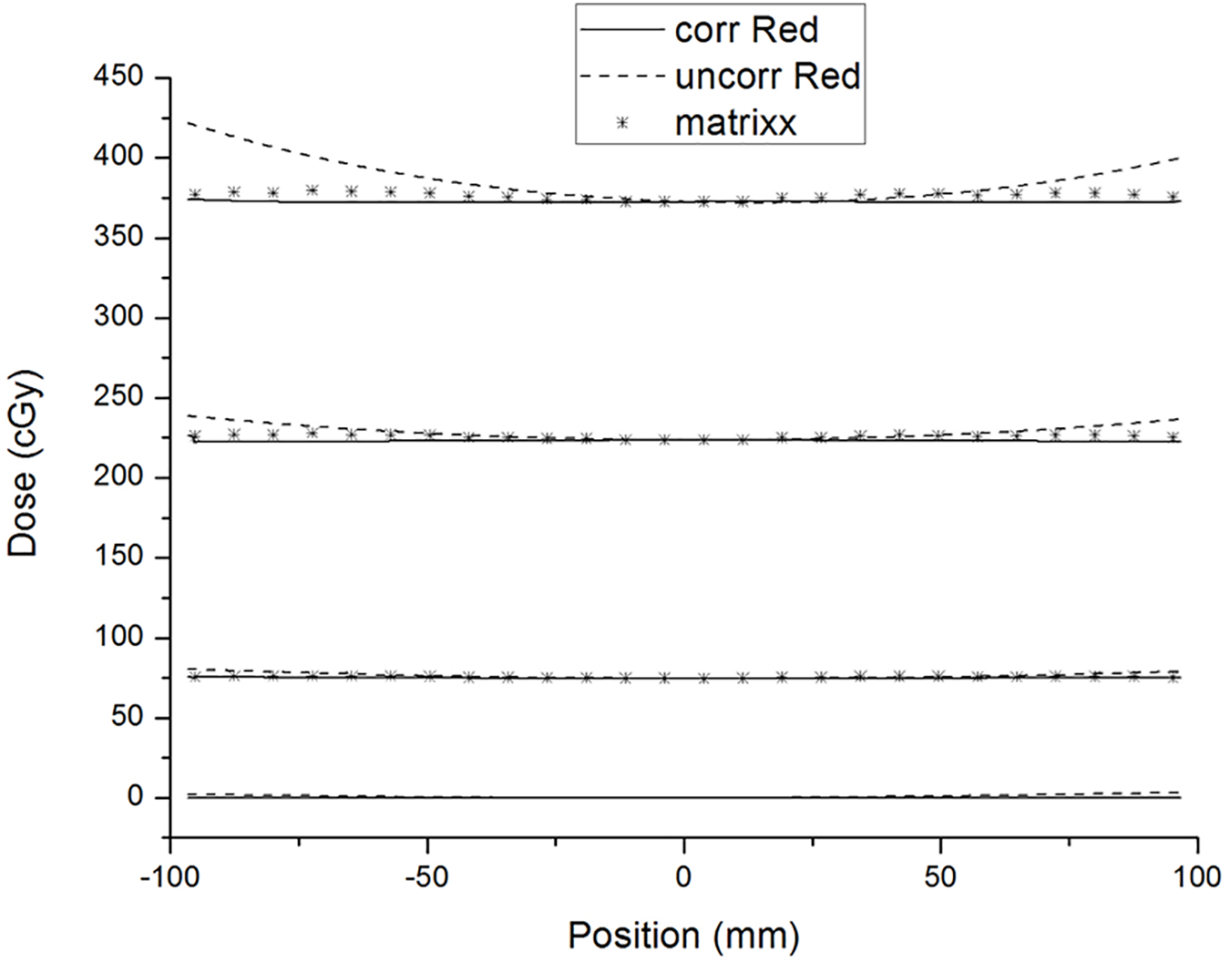
Comparison of the dose values obtained from the film of without lateral effect correction (dashed lines) and with lateral effect correction (solid lines) in the red channel. The dose values obtained using Matrixx array are also presented.

Fig 2 shows the scanned pixel values of the red channel and their second-order polynomial fits at different constant dose values. The range of the parabolic symmetry axis is from x = 4.9 mm to x = 9.6 mm. The parameter b = 6.7 mm was used for Eq (3). The relationships between the parameters a and c in the RGB channels are shown in Fig 3. Fig 4 shows the results of lateral effect correction for the red channel. The dose values obtained (Eq 5) with and without the application of lateral effect correction for various doses (0, 74.5, 223.5 and 372.5 cGy) and the results measured using Matrixx array are shown. The effect of the correction is clearly visible in the figure. Similar results were obtained for the green and blue channels (see S1 and S2 Figs). The uncertainty of RGB channels for before and after lateral effect correction were shown in Table 1.

**Table 1:** Before and after correction of lateral effect (consider dose and position dependence), the mean ± 2SD of RGB channels were provided.

Dose (cGy)	Red channel		Green channel		Blue channel	
	Corr	Uncorr	Corr	Uncorr	Corr	Uncorr
74.5	74.8±0.6	76.2±3.2	74.5±0.2	76.6±4.2	74.9±1.0	79.7±10.0
223.5	223.1±0.9	228.2±8.5	223.2±0.2	227.4±7.5	222.5±0.3	229.0±13.2
372.5	372.4±0.6	384.9±25.7	371.8±0.2	380.7±17.7	372.7±0.9	384.8±23.0

### Verification of dose calibration accuracy

**Fig 5:**
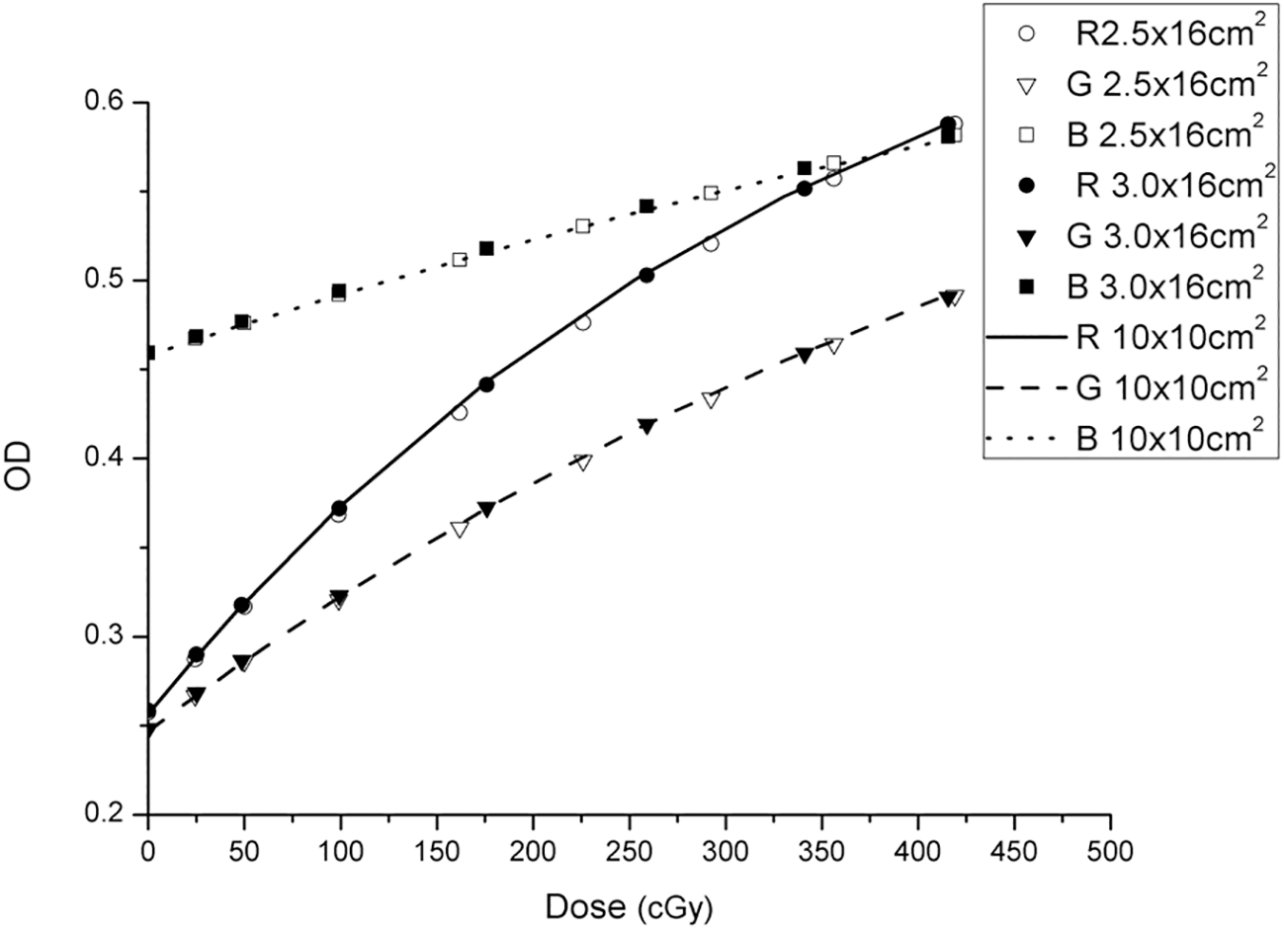
RGB calibration curves obtained using the three methods. R, G and B represent red, green and blue, respectively.

For each calibration method, the average OD values of three times measurement were used for the calibration, and the uncertainty of OD between each measurement was <0.8%. The RGB calibration curves obtained using the three calibration methods are shown in Fig 5. Compared with the square-field calibration method, the maximum deviations of the results using the 3 × 16 and 2.5 × 16 cm^2^ strips were 1.1% and 2.9%, respectively.

### Triple-channel correction and dose conversion

**Fig 6:**
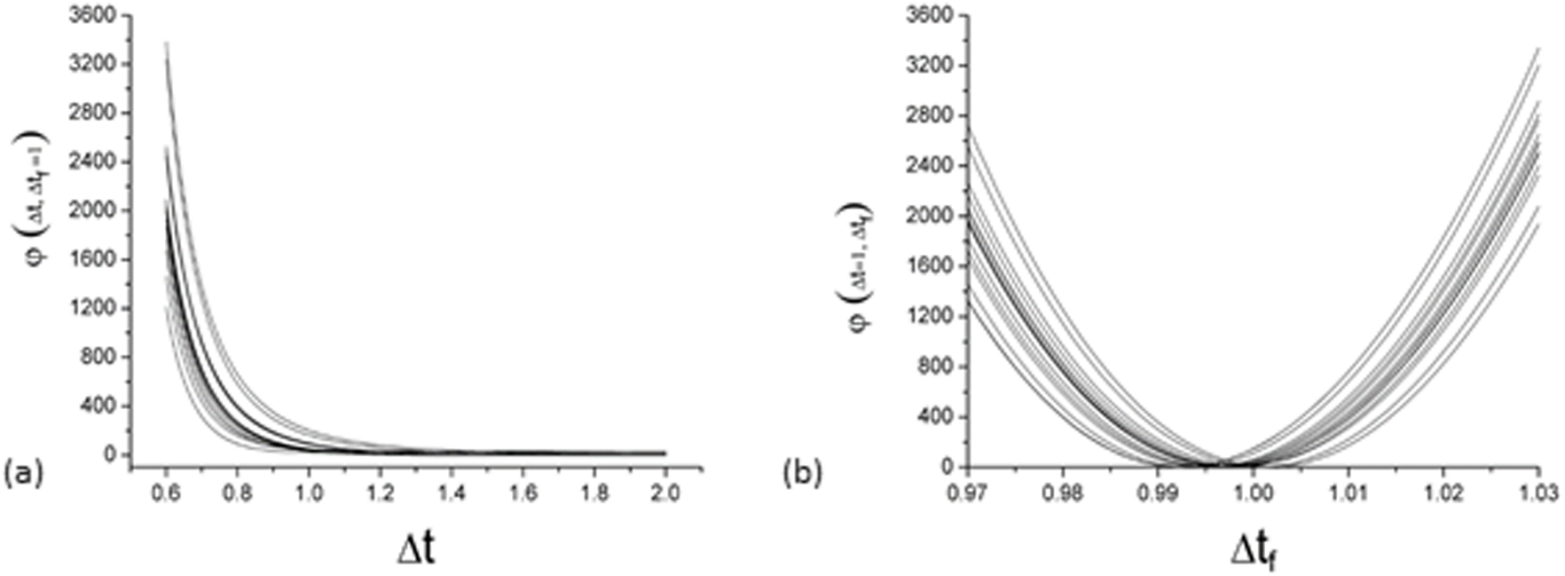
*φ*(Δ*t*,Δ*t*_*f*_) of 15 separate pixels in a profile changing with Δ*t*_*f*_ or Δ*t*; *φ*(Δ*t*,Δ*t*_*f*_ = 1) (a) and *φ*(Δ*t* = 1,Δ*t*_*f*_) (b).

**Fig 7:**
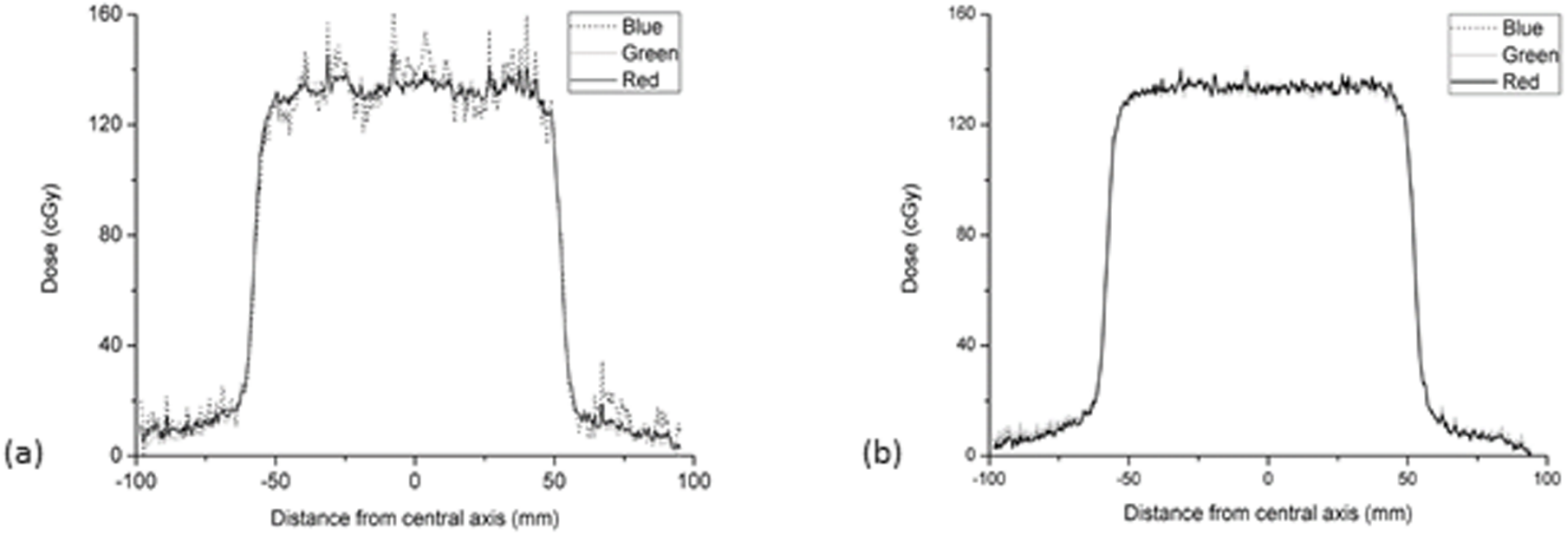
Profiles obtained from single- (a) and triple-channel (b) dose conversions.

A set of optimal Δ*t* and Δ*t*_*f*_ values was sought to minimize the function *φ*(Δ*t*,Δ*t*_*f*_), but only an optimal Δ*t*_*f*_ was found in this process. Fig 6 shows an example of this result; the *φ*(Δ*t*,Δ*t*_*f*_ = 1) and *φ*(Δ*t* = 1,Δ*t*_*f*_) of 15 separate pixels in a profile changing with Δ*t* and Δ*t*_*f*_ are shown in Fig 6A and 6B, respectively. It can be seen that there is a minimal value in Fig 6B and no minimal value in Fig 6A. Because of the absence of an optimal Δ*t* to minimize *φ*(Δ*t*,Δ*t*_*f*_), we set Δ*t* = 1 and only found an optimal Δ*t*_*f*_. Fig 7A shows the profiles obtained from single-channel dose conversions; because of the interference caused by differences in relative thickness, the results of single-channel dose conversions show different degrees of disturbance. Within the central 80% of the field width, the mean ± 2 SD of Red, Green and Blue single channels were 134.1±2.2, 133.6±2.9 and 135.5±7.9 cGy, respectively. Fig 7B shows the triple-channel dose conversion profiles for *φ*(Δ*t* = 1,Δ*t*_*f*_), where the disturbances had been obviously reduced, the mean ± 2 SD of RGB channels were 133.7±1.6, 133.1±1.9 and 133.5±1.7 cGy, respectively. That is to say, the uncertainty of RGB triple-channels were less than 1%.

### Procedure comparison

**Fig 8:**
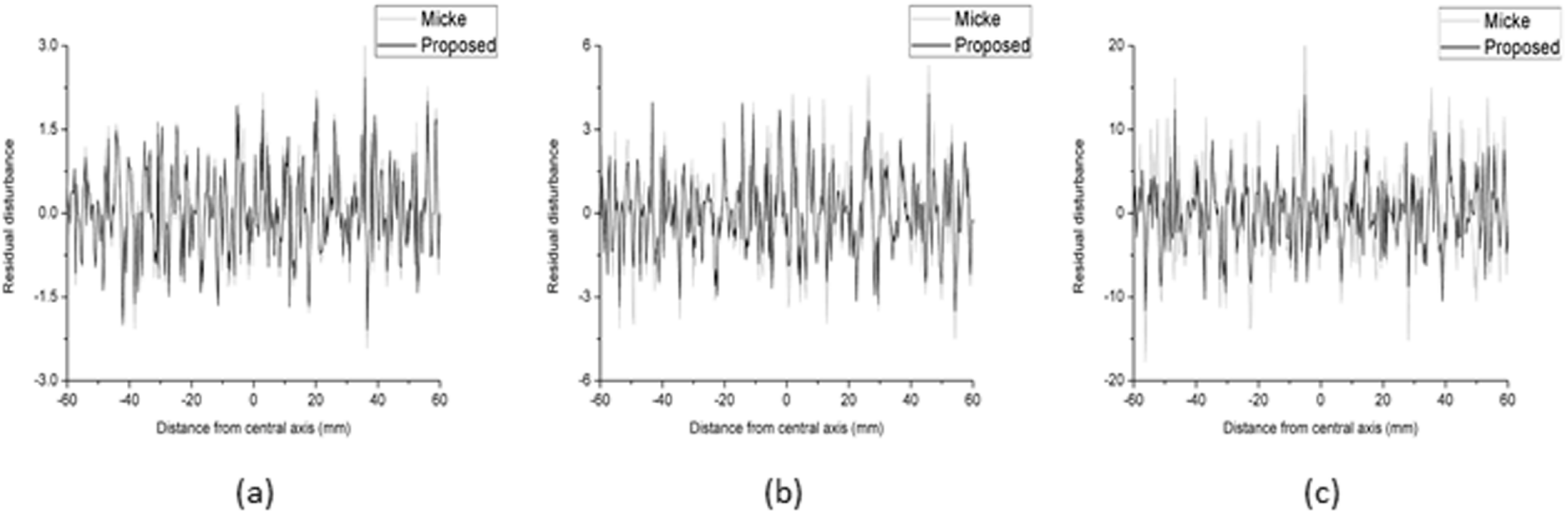
Comparison of residual disturbances between the proposed method and the method of Micke A et al. for doses of 48 (a), 176 (b) and 415 (c) cGy.

**Fig 9:**
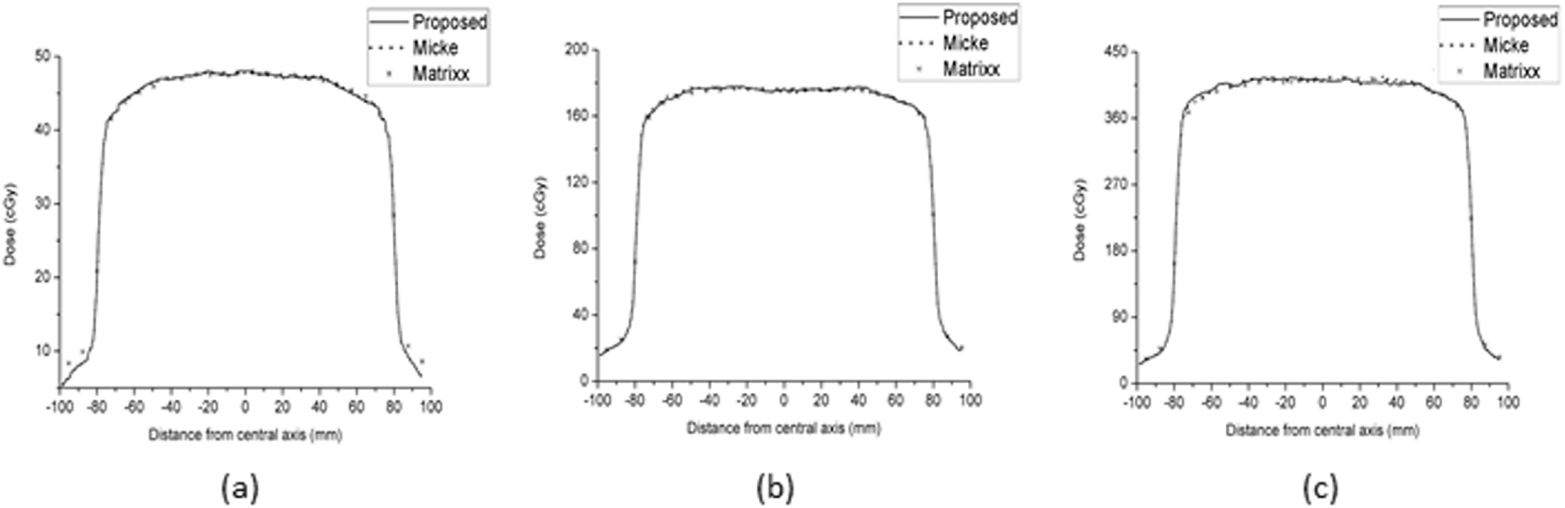
Comparison of the dose profiles among the proposed method (solid lines), the method of Micke A et al. (dotted lines) and the Matrixx results (*) for doses of 48 (a), 176 (b) and 415 (c) cGy.

Fig 8 shows the comparison of the residual disturbances between the proposed triple-channel method and the method of Micke A et al. [11] at three dose levels (48, 176 and 415 cGy). To analyse the results of Micke A et al., FILMQA PRO software was used, and the dose maps were exported as comma-separated values. The residual disturbances were extracted from the dose profile via median smoothing. The results show that the residual disturbance ranges of the proposed method are smaller than those of Micke A et al. by 5.3%, 20.9% and 31.4% at the three dose levels. Fig 9 shows the comparison of the dose profiles among the proposed method, the method of Micke A et al. and the Matrixx results.

### Comparisons of PDD and OARs between the EBT3 and ionization chamber results

**Fig 10:**
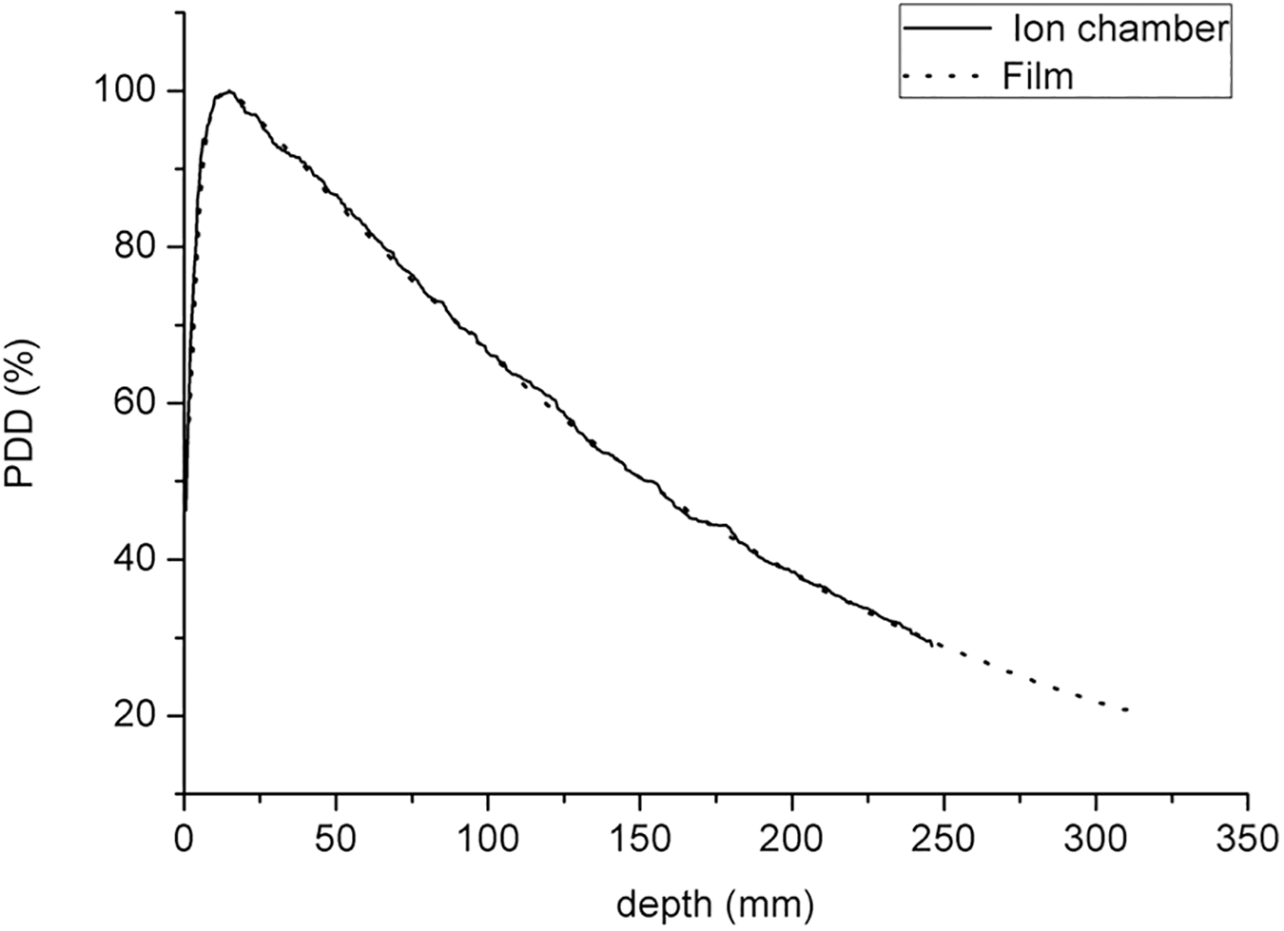
Beam of 10 × 10 cm2 and SSD = 100 cm. Comparison of measured PDDs between the ionization chamber scanning data and the EBT3 film data.

Fig 10 shows the comparison of PDD between the present method and ionization chamber. The PDDs are normalized to the maximum dose depth (d_max_). The d_max_ and the PDD at depths of 10 and 20 cm, as measured by EBT3 film dosimetry, were 1.47 cm, 66.6% and 38.4%, respectively. Compared with the data obtained using the ionization chamber (1.4 cm, 66.4% and 38.1%), the differences were 0.07 cm, 0.3% and 0.8%, respectively.

**Fig 11:**
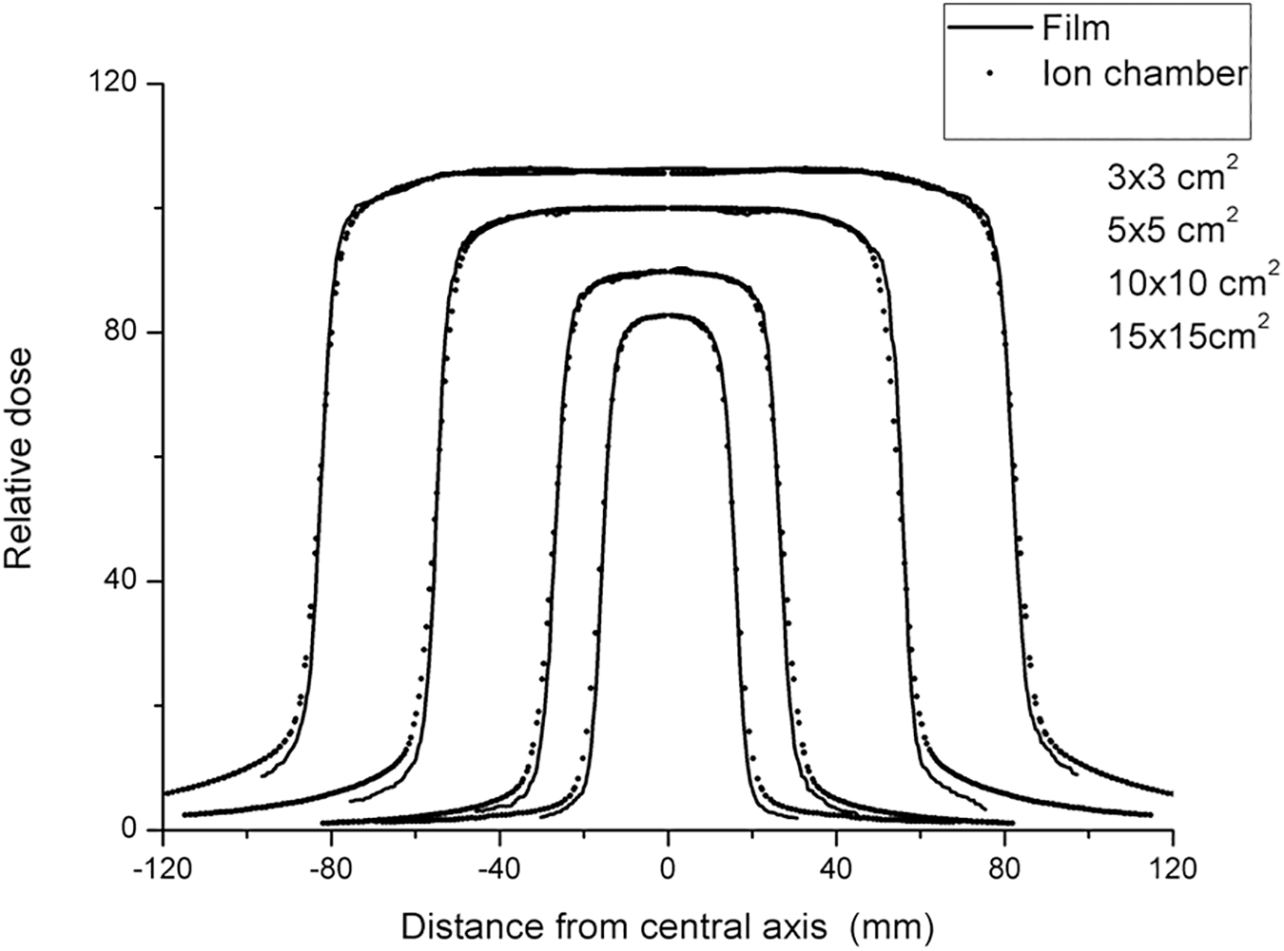
SSD = 100 cm. Comparison of the relative dose profiles between the EBT3 film and ionization chamber data.

Fig 11 shows the comparison of OARs between the present method and ionization chamber, in which the OARs are normalized to a 10 × 10-cm^2^ field. Within the central 80% of the field width, the OARs obtained using the two measurement tools are in agreement. The flatness and symmetry results, obtained according to the International Atomic Energy Agency (IAEA) protocol [31], are shown in Table 2.

**Table 2:** Comparison of flatness and symmetry for the results obtained using the EBT3 film and ionization chamber.

	EBT3 film		Ionization chamber	
	Flatness (%)	Symmetry (%)	Flatness (%)	Symmetry (%)
3 × 3 cm^2^	8.30	0.63	8.90	0.83
5 × 5 cm^2^	3.86	0.76	4.90	0.63
10 × 10 cm^2^	2.05	0.65	1.83	0.49
15 × 15 cm^2^	1.96	0.71	1.71	0.75

### IMRT verifications

**Fig 12:**
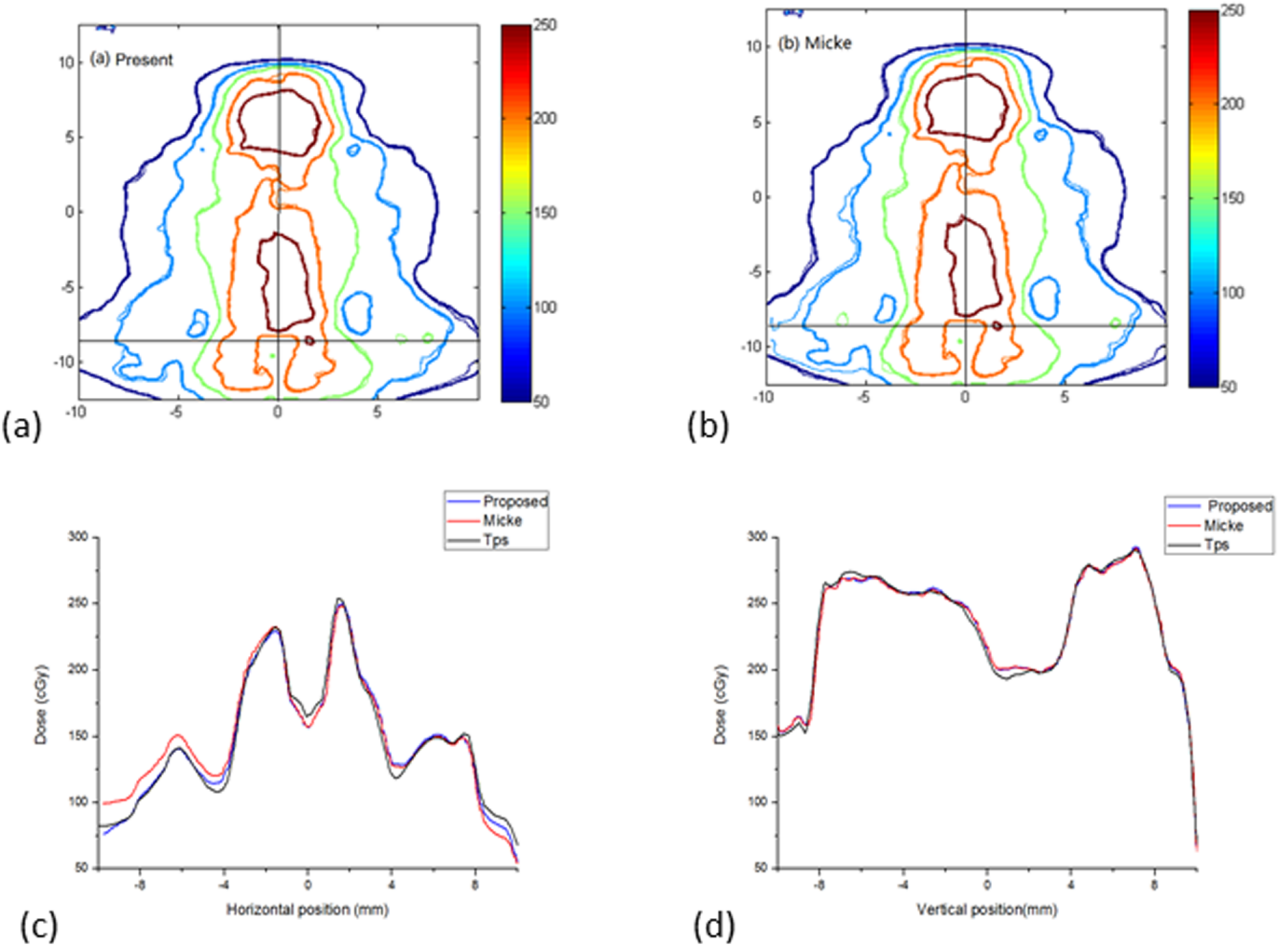
Comparison of isodose maps for a large field IMRT plan. (a) Isodose maps of the present method, (b) isodose maps of the method of Micke et al.; (c) and (d) show horizontal and vertical profiles.

Table 3 shows the γ 2D(3%/3 mm), γ2D(2%/2 mm) and γ 2D(1%/1 mm) pass rates between the film dose distributions and the planning system. Compared with Micke A et al., the gamma pass rate improved by an average of 3% with the criteria 1%/1 mm for small fields, and there were no differences in results with the criteria 3%/3 mm and 2%/2 mm. The gamma pass rate improved by an average of 1% with the criteria 2%/2 mm and 5% with the criteria 1%/1 mm for medium fields, and there was no difference with the criteria 3%/3 mm. The gamma pass rate improved by an average of 2% with the criteria 3%/3 mm, 3% with the criteria 2%/2 mm and 8% with the criteria 1%/1 mm for large fields. Fig 12 shows a comparison of the data from the large IMRT field, and Fig 12A and 12B show the isodose maps for the same IMRT field based on the method present here and the method of Micke et al., respectively. The horizontal and vertical profiles (corresponding to the crosshairs in the isoline maps) are shown in Fig 12C and 12D. In Fig 12C, the profile of TPS was closer to the profile of combined method, this results demonstrates the necessity of lateral effect correction before triple-channel analysis. In Fig 12D, there is almost no difference in the profile of the two triple-channel methods.

**Table 3:** Comparisons of gamma passing rates between the films measured and the TPS dose maps for the small (s), medium (M), large (L) and total (T) IMRT plan fields.

	The present method			Mike et al. method		
	3%/3mm	2%/2mm	1%/1mm	3%/3mm	2%/2mm	1%/1mm
Mean(S) ± SD	99.9±0.2	98.5±1.8	86.2±7.5	99.9±0.2	98.3±2.0	82.9±11.1
Mean(M) ±SD	98.9±1.8	96.5± 3.8	78.0± 11.3	98.7± 1.9	95.2± 5.4	72.5± 11.5
Mean(L) ± SD	97.4± 2.6	92.8± 6.3	72.2± 11.1	95.4± 3.5	90.0± 4.3	64.4±8.2
Mean(T) ± SD	98.7± 2.0	95.9± 4.8	78.8± 11.7	98.0± 2.9	94.5± 5.2	73.3± 12.4

## Discussion

The single-film exposure method can reduce irradiation times (~5 min) and possible errors related to phantom set-up and is easy to perform. It is more convenient and economical than the square-field calibration method. Menegotti L et al. [21] proposed a single-film exposure method with a 3.6-cm strip size and six irradiation dose levels. However, Mendez I et al. [32] indicated that the accuracy of the calibration curve could be reduced only six dose levels are used. To increase the dose levels, smaller strips should be used, but two important factors should be considered: (1) the positional accuracy of the linac jaws and (2) the smallest sensitive size of the ionization chamber. The calibration ionization chamber used here was a Semiflex ionization chamber (31010), for which the minimum field size recommended by the manufacturer is 3 × 3 cm^2^. A strip size of 3 cm and irradiation at seven dose levels were found to be suitable. Although we tried to ensure the dose uniformity of each strip, there was an absorbed dose gradient (variation of 10%) in each strip. Therefore, to reduce the uncertainty of the film dose calibration, it is necessary to ensure precise ionization chamber position set-up and mark the centre of each film strip. Errors in ionization chamber position set-up were less than ± 1 mm (uncertainty < 0.5%). The films were marked prior to irradiation to indicate the centre position of each strip according to the linac crosshairs and a large sheet of graph paper (placed under the film).

To reduce the effect of issues related environmental and scanner variability for film uniformity, the scanner temperature in our experiment was 20 ± 2°C, and all films were scanned within 30 min. Recently, a new and efficient protocol for radiochromic film dosimetry using EBT3 films was published [12] that combines calibration and measurement in a single scan, and issues related to environmental and scanner variability can be eliminated using this protocol. The optimal application of the protocol was studied by Marrazzo et al. [33], and the effects of environmental and scanner variability were negligible.

The method for lateral effect correction referenced in the study by Poppinga D et al. [22] was used prior to triple-channel dose analysis. As shown in Fig 4, after correction for lateral effect, the trend of dose profiles were close to the results of Matrixx array. The range of uncertainty (SD) from 3.2–25.7 cGy decreased to 0.2–1.0 cGy, as shown in Table 1. The measurement of the curvature factor can be obtained in combination with our single-film calibration method. We also considered that to only correct the position dependence of the lateral effect, the curvature factor of the lateral effect is assumed to be constant and could be obtained from a non-irradiated film. Using this method, the uncertainty of lateral effect for dose range from 0–223.5 cGy can be reduced to 0.3–2.4cGy. But the effect of lateral effect correction for only correct the position dependence is poor at higher dose range (greater than 372.5 cGy) (see S3 and S5 Figs and S1 Table).

The advantages of correcting lateral effects on measurements may not be as great as those associated with the PDD if the depth direction is not properly aligned with the scanning direction. However, when films were used to check other data, such as IMRT QA and profile data in two directions that are not aligned with the scanning direction, lateral effect corrections can provide a greater advantage.

In this study, we established a proposed triple-channel dose analysis model based on the method of Micke A et al. [11]. We divided the film into two components (active and inactive layers), as opposed to considering only the active layer as was done by Micke A et al [11]. The optimal target function of the triple-channel model *φ*(Δ*t*,Δ*t*_*f*_) has two parameters, Δ*t* and Δ*t*_*f*_. When Δ*t*_*f*_ = Δ*t*, which indicates that the film is only composed of an active layer, the optimal target function will be the same as that proposed by Micke A et al [11]. if the same form of calibration function was used. The initial purpose was to explore the general form of *φ*(Δ*t*,Δ*t*_*f*_). However, it was unusual to find that there was no optimal Δ*t* to minimize the function *φ*(Δ*t*,Δ*t*_*f*_) when Δ*t* ranged from approximately 0.6 to 2.0. Because of the absence of an optimal Δ*t* to minimize *φ*(Δ*t*,Δ*t*_*f*_), we set Δ*t* = 1 and only found an optimal Δ*t*_*f*_ for the calculated dose. This method shows a certain degree of improvement in the residual disturbance, which can be observed in Fig 8. A more detailed study on the nonuniformity of the inactive layer should be conducted.

The different sizes of IMRT fields were verified to evaluate the possible real benefit of our present method. For small IMRT fields, because the dose distributions were concentrated in the centre of the film, the lateral effect was very small, and the difference between the results of the two methods was mainly due to differences in the triple-channel methods. However, there was almost no difference in the results of the two methods, meaning that the two triple-channel methods are not fundamentally different in the IMRT results they produce. The similar conclusion can be obtained from Fig 12D. The differences in the IMRT results of the two methods increased with increasing field size. The gamma pass rate showed an approximately 2% improvement for large IMRT fields with the criteria 3%/3 mm, that is because the lateral effect also be corrected in present method.

Research on lateral effects and film inhomogeneity is not new, and many studies have demonstrated approaches the can reduce the influences of lateral effects or film inhomogeneity [11,21–27]. In this study, only the triple-channel analysis part shows a small innovation in the methodology; the present method is based on the methods of Poppinga D et al. [22] and Micke et al. [11], these two methods have proved to be very effective for scanner lateral effect and film inhomogeneity corrections, respectively. In our results, the verification of IMRT plans shows that is necessity to combine corrections of lateral effects and the influence of film inhomogeneity, especially for large IMRT fields, such those used in treating nasopharyngeal cancer and lung cancer.

## Conclusions

A complete set of methods for radiochromic film dosimetry, including calibration and correction for lateral effects and film inhomogeneity, is presented. The results of the IMRT verification demonstrate the necessary of correcting for both lateral effects and film inhomogeneity. The uncertainty of film results can be reduced to 1%.

## Supporting information

S1 Table Before and after correction of lateral effect (consider dose and position dependence), the mean ± 2SD of RGB channels were provided. (DOCX)

S1 Fig Comparison of the dose values obtained from the film of without (dashed lines) and with (solid lines) lateral effect correction (consider dose and position dependence) in the green channel. The dose values obtained using Matrixx array are also presented.
(TIF)

S2 Fig Comparison of the dose values obtained from the film of without (dashed lines) and with (solid lines) lateral effect correction (consider dose and position dependence) in the blue channel. The dose values obtained using Matrixx array are also presented.
(TIF)

S3 Fig Comparison of the dose values obtained from the film of without (dashed lines) and with (solid lines) lateral effect correction (only consider position dependence) in the red channel. The dose values obtained using Matrixx array are also presented.
(TIF)

S4 Fig Comparison of the dose values obtained from the film of without (dashed lines) and with (solid lines) lateral effect correction (only consider position dependence) in the green channel. The dose values obtained using Matrixx array are also presented.
(TIF)

S5 Fig Comparison of the dose values obtained from the film of without (dashed lines) and with (solid lines) lateral effect correction (only consider position dependence) in the blue channel. The dose values obtained using Matrixx array are also presented.
(TIF)

## References

[1] Klein EE, Hanley J, Bayouth J, Yin F-F, Simon W, Dresser S, et al Task Group 142 report: Quality assurance of medical accelerators. Medical Physics. 2009;36:4197 doi: 10.1118/1.3190392 19810494 10.1118/1.3190392

[2] Niroomand-Rad A, Blackwell CR, Coursey BM, Gall KP, Galvin JM, McLaughlin WL, et al Radiochromic film dosimetry: Recommendations of AAPM Radiation Therapy Committee Task Group 55. Medical Physics. 1998;25:2093 doi: 10.1118/1.598407 9829234 10.1118/1.598407

[3] Lindsay P, Rink A, Ruschin M, and Jaffray D. “Investigation of energy dependence of EBT and EBT-2 Gafchromic film,” Medical Physics.2010;37:571 doi: 10.1118/1.3291622 20229865 10.1118/1.3291622

[4] Barbeiro AR, Ureba A, Baeza JA, Linares R, Perucha M, JimeÂnez-Ortega E, et al 3D VMAT Verification Based on Monte Carlo Log File Simulation with Experimental Feedback from Film Dosimetry. PLoS ONE.2016; 11(11): e0166767 doi: 10.1371/journal.pone.0166767 27870878 10.1371/journal.pone.0166767 PMC5117721

[5] Yusof FH, Ung NM, Wong JHD, Jong WL, Ath V, Phua VCE, et al (2015) On the Use of Optically Stimulated Luminescent Dosimeter for Surface Dose Measurement during Radiotherapy. PLoS ONE,2015; 10(6): e0128544 doi: 10.1371/journal.pone.0128544 26052690 10.1371/journal.pone.0128544 PMC4459977

[6] Fuss Martina, Sturtewagen Eva, Carlos DeWagter Dietmar Georg. Dosimetric characterization of GafChromic EBT film and its implication on film dosimetry quality assurance. Physics in medicine and biology. 2007; 52:4211–25. doi: 10.1088/0031-9155/52/14/013 17664604 10.1088/0031-9155/52/14/013

[7] Mack A, Mack G, Weltz D, Scheib SG, Böttcher HD, Seifert V. High precision film dosimetry with GAFCHROMIC films for quality assurance especially when using small fields. Medical Physics. 2003; 30:2399–409. doi: 10.1118/1.1593634 14528962 10.1118/1.1593634

[8] Ferreira BC, Lopes MC, Capela M. Evaluation of an Epson flatbed scanner to read Gafchromic EBT films for radiation dosimetry. Physics in medicine and biology. 2009;54:1073–85. doi: 10.1088/0031-9155/54/4/017 19168937 10.1088/0031-9155/54/4/017

[9] Anna Gueli, Nina Cavalli, De Vincolis Renato, Luigi Raffaele, Troja Sebastiano O. Background fog subtraction methods in Gafchromic® dosimetry. Radiation Measurements. 2015;72:L44–52.

[10] Lynch BD, Kozelka J, Ranade MK, Li JG, Simon WE, Dempsey JF. Important considerations for radiochromic film dosimetry with flatbed CCD scanners and EBT GAFCHROMIC film. Medical Physics. 2006;33:4551 doi: 10.1118/1.2370505 17278806 10.1118/1.2370505

[11] Micke A, Lewis DF, Yu X. Multichannel film dosimetry with nonuniformity correction. Medical Physics. 2011;38:2523 doi: 10.1118/1.3576105 21776787 10.1118/1.3576105

[12] Lewis D, Micke A, Yu Xiang, Chan Maria F. An efficient protocol for radiochromic film dosimetry combining calibration and measurement in a single scan. Medical Physics 39, 6339 (2012); doi: 10.1118/1.4754797 23039670 10.1118/1.4754797 PMC9381144

[13] Kairn T, Aland T, Kenny J. Local heterogeneities in early batches of EBT2 film: a suggested solution. Physics in medicine and biology. 2010;55:L37–42. doi: 10.1088/0031-9155/55/15/L02 20616403 10.1088/0031-9155/55/15/L02

[14] McCaw TJ, Micka JA, Dewerd LA. Characterizing the marker-dye correction for Gafchromic((R)) EBT2 film: a comparison of three analysis methods. Medical Physics.2011;38:5771–7. doi: 10.1118/1.3639997 21992391 10.1118/1.3639997

[15] Devic S, Tomic N, Soares CG, Podgorsak EB. Optimizing the dynamic range extension of a radiochromic film dosimetry system. Medical Physics. 2009;36:429 doi: 10.1118/1.3049597 19291981 10.1118/1.3049597

[16] Dreindl R, Georg D, Stock M. Radiochromic film dosimetry: considerations on precision and accuracy for EBT2 and EBT3 type films. Zeitschrift fur medizinische Physik. 2014;24:153–63. doi: 10.1016/j.zemedi.2013.08.002 24055395 10.1016/j.zemedi.2013.08.002

[17] Andrés C, del Castillo A, Tortosa R, Alonso D, Barquero R. A comprehensive study of the Gafchromic EBT2 radiochromic film. A comparison with EBT. Medical Physics. 2010;37:6271 doi: 10.1118/1.3512792 21302783 10.1118/1.3512792

[18] Aland T, Kairn T, Kenny J. Evaluation of a Gafchromic EBT2 film dosimetry system for radiotherapy quality assurance. Australasian physical & engineering sciences in medicine / supported by the Australasian College of Physical Scientists in Medicine and the Australasian Association of Physical Sciences in Medicine. 2011;34:251–60. 10.1007/s13246-011-0072-6 21465275

[19] Richley L, John AC, Coomber H, Fletcher S. Evaluation and optimization of the new EBT2 radiochromic film dosimetry system for patient dose verification in radiotherapy. Physics in medicine and biology. 2010;55:2601–17. doi: 10.1088/0031-9155/55/9/012 20393235 10.1088/0031-9155/55/9/012

[20] Saur S, Frengen J. GafChromic EBT film dosimetry with flatbed CCD scanner: A novel background correction method and full dose uncertainty analysis. Medical Physics. 2008;35:3094. 10.1118/1.2938522 18697534

[21] Menegotti L, Delana A, Martignano A. Radiochromic film dosimetry with flatbed scanners: A fast and accurate method for dose calibration and uniformity correction with single film exposure. Medical Physics. 2008;35:3078. 10.1118/1.2936334 18697531

[22] Poppinga D, Schoenfeld AA, Doerner KJ, Blanck O, Harder D, Poppe B. A new correction method serving to eliminate the parabola effect of flatbed scanners used in radiochromic film dosimetry. Medical Physics. 2014;41:021707 doi: 10.1118/1.4861098 24506598 10.1118/1.4861098

[23] Wen N, Lu S, Kim J, Qin Y, Huang Y, Zhao B, Liu C, Chetty IJ. Precise film dosimetry for stereotactic radiosurgery and stereotactic body radiotherapy quality assurance using Gafchromic™ EBT3 films. Radiation Oncology. 2016; 11:132 doi: 10.1186/s13014-016-0709-4 27716323 10.1186/s13014-016-0709-4 PMC5050597

[24] Hartmann B, Martišíková Mr, Jäkel O. Technical Note: Homogeneity of Gafchromic® EBT2 film. Medical Physics. 2010;37:1753 doi: 10.1118/1.3368601 20443496 10.1118/1.3368601

[25] Mendez I, Peterlin P, Hudej R, Strojnik A, Casar B. On multichannel film dosimetry with channel-independent perturbations. Medical Physics.2014;41:011705 doi: 10.1118/1.4845095 24387497 10.1118/1.4845095

[26] Mayer RR, Ma F, Chen Y, Miller RI, Belard A, McDonough J, et al Enhanced dosimetry procedures and assessment for EBT2 radiochromic film. Medical Physics.2012;39:2147–55. doi: 10.1118/1.3694100 22482635 10.1118/1.3694100

[27] Perez Azorin JF, Ramos Garcia LI, Marti-Climent JM. A method for multichannel dosimetry with EBT3 radiochromic films. Medical Physics.2014;41:062101 doi: 10.1118/1.4871622 24877828 10.1118/1.4871622

[28] Low Daniel A., Moran Jean M., et al Task Group 120 report: Dosimetry tools and techniques for IMRT. Medical Physics. 2011; 38:1313 doi: 10.1118/1.3514120 21520843 10.1118/1.3514120

[29] Tamponi M, Bona R, Poggiu A, Marini P. A practical tool to evaluate dose distributions using radiochromic film in radiation oncology. Physica medica.2015;31:1–6. 25113942 10.1016/j.ejmp.2014.07.009

[30] Paelinck L et al Precautions and strategies in using a commercial flatbed scanner for radiochromic film dosimetry. Phys. Med. Biol. 52 (2007) 231–242. doi: 10.1088/0031-9155/52/1/015 17183138 10.1088/0031-9155/52/1/015

[31] Podgorsak EB, Andreo P, Evans MDC, Hendry JH, Horton JL. Radiation Oncology Physics:A handbook for teachers and students.2005.

[32] Mendez I, Hartman V, Hudej R, Strojnik A, Casar B. Gafchromic EBT2 film dosimetry in reflection mode with a novel plan-based calibration method. Medical Physics.2013;40:011720 doi: 10.1118/1.4772075 23298090 10.1118/1.4772075

[33] Marrazzo L, Zani M, Pallotta S, Arilli C, Casati M, Compagnucci A, Talamonti C, Bucciolini M. GafChromic(®) EBT3 films for patient specific IMRT QA using a multichannel approach. Physica medica.2015;31:1035–42. doi: 10.1016/j.ejmp.2015.08.010 26429383 10.1016/j.ejmp.2015.08.010

