# Supplementary material for: The combination of the error correction methods of GAFCHROMIC EBT3 film
Source: PLoS One. 2017 Jul 27;12(7):e0181958. doi: 10.1371/journal.pone.0181958 (PMC5531657; doi:10.1371/journal.pone.0181958)
Supplement: S1 Table — (DOCX) [file pone.0181958.s001.docx]

**S1 Table.** Before and after correction of lateral effect (consider dose and position dependence), the mean ± 2SD of RGB channels were provided.

| Dose (cGy) | Red channel | |  | Green channel | |  | Blue channel | |
| --- | --- | --- | --- | --- | --- | --- | --- | --- |
|  | Corr | Uncorr |  | Corr | Uncorr |  | Corr | Uncorr |
| 74.5 | 74.8±0.5 | 76.2±3.2 |  | 74.5±0.3 | 76.6±4.2 |  | 75.1±2.1 | 79.7±10.0 |
| 223.5 | 224.7±2.4 | 228.2±8.5 |  | 223.9±1.4 | 227.4±7.5 |  | 222.5±2.4 | 229.0±13.2 |
| 372.5 | 377.9±12.0 | 384.9±25.7 |  | 374.8±5.1 | 380.7±17.7 |  | 375.6±6.9 | 384.8±23.0 |
