# Supplementary figures and images for: The combination of the error correction methods of GAFCHROMIC EBT3 film
Source: PLoS One. 2017 Jul 27;12(7):e0181958. doi: 10.1371/journal.pone.0181958 (PMC5531657; doi:10.1371/journal.pone.0181958)

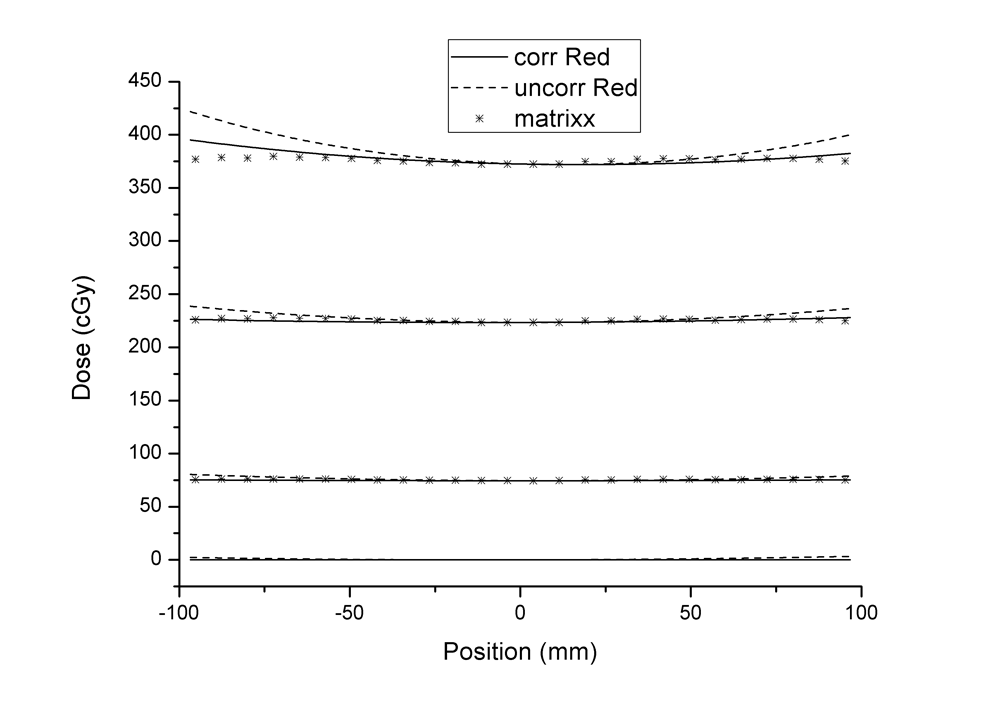

Supplement: S3 Fig — The dose values obtained using Matrixx array are also presented. (TIF) [file pone.0181958.s004.tif]

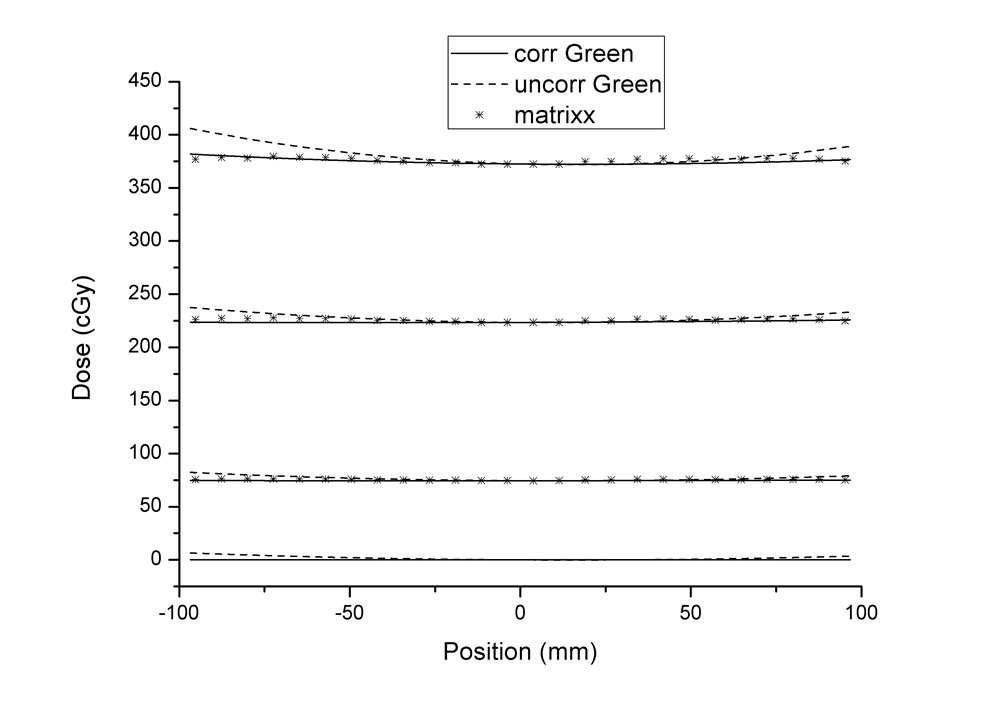

Supplement: S4 Fig — The dose values obtained using Matrixx array are also presented. (TIF) [file pone.0181958.s005.tif]

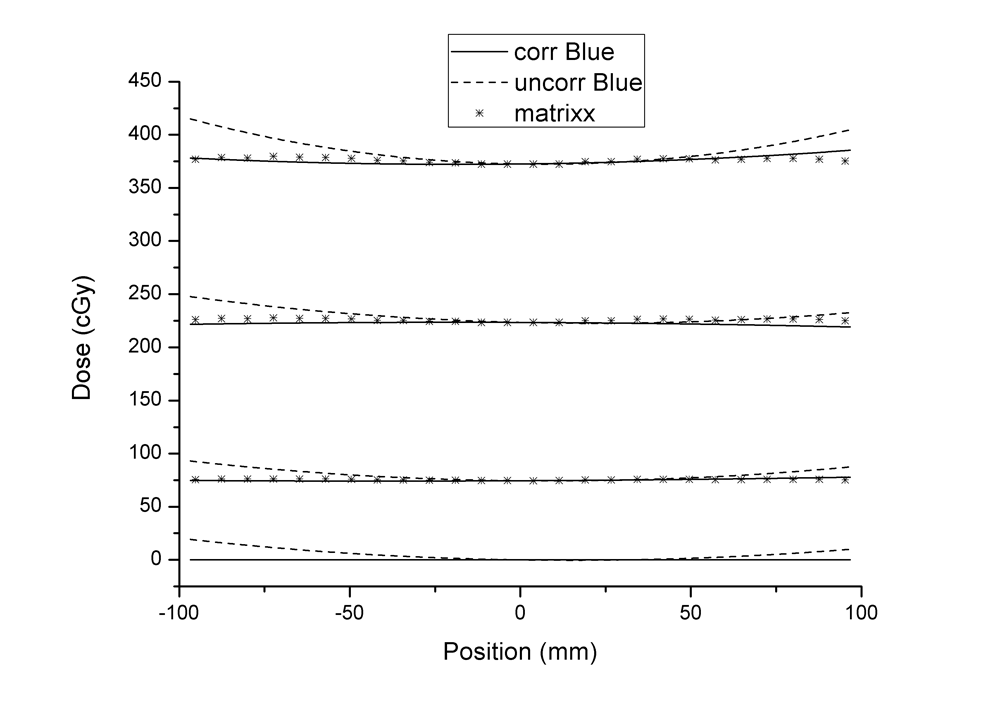

Supplement: S5 Fig — The dose values obtained using Matrixx array are also presented. (TIF) [file pone.0181958.s006.tif]
